# Expression of socially sensitive genes: The multi-ethnic study of atherosclerosis

**DOI:** 10.1371/journal.pone.0214061

**Published:** 2019-04-11

**Authors:** Kristen M. Brown, Ana V. Diez-Roux, Jennifer A. Smith, Belinda L. Needham, Bhramar Mukherjee, Erin B. Ware, Yongmei Liu, Steven W. Cole, Teresa E. Seeman, Sharon L. R. Kardia

**Affiliations:** 1 Department of Epidemiology, School of Public Health, University of Michigan, Ann Arbor, Michigan, United States of America; 2 Department of Epidemiology, Rollins School of Public Health, Emory University, Atlanta, Georgia, United States of America; 3 Department of Epidemiology, Dornsife School of Public Health, Drexel University, Philadelphia, Pennsylvania, United States of America; 4 Survey Research Center, Institute for Social Research, University of Michigan, Ann Arbor, Michigan, United States of America; 5 Department of Biostatistics, School of Public Health, University of Michigan, Ann Arbor, Michigan, United States of America; 6 Department of Epidemiology and Prevention, Wake Forest University, Winston-Salem, North Carolina, United States of America; 7 Department of Medicine, Division of Hematology-Oncology, University of California-Los Angeles, Los Angeles, California, United States of America; 8 Department of Medicine, University of California-Los Angeles, Los Angeles, California, United States of America; Mayo Clinic Arizona, UNITED STATES

## Abstract

**Background:**

Gene expression may be an important biological mediator in associations between social factors and health. However, previous studies were limited by small sample sizes and use of differing cell types with heterogeneous expression patterns. We use a large population-based cohort with gene expression measured solely in monocytes to investigate associations between seven social factors and expression of genes previously found to be sensitive to social factors.

**Methods:**

We employ three methodological approaches: 1) omnibus test for the entire gene set (Global ANCOVA), 2) assessment of each association individually (linear regression), and 3) machine learning method that performs variable selection with correlated predictors (elastic net).

**Results:**

In global analyses, significant associations with the a priori defined socially sensitive gene set were detected for major or lifetime discrimination and chronic burden (p = 0.019 and p = 0.047, respectively). Marginally significant associations were detected for loneliness and adult socioeconomic status (p = 0.066, p = 0.093, respectively). No associations were significant in linear regression analyses after accounting for multiple testing. However, a small percentage of gene expressions (up to 11%) were associated with at least one social factor using elastic net.

**Conclusion:**

The Global ANCOVA and elastic net findings suggest that a small percentage of genes may be “socially sensitive,” (i.e. demonstrate differential expression by social factor), yet single gene approaches such as linear regression may be ill powered to capture this relationship. Future research should further investigate the biological mechanisms through which social factors act to influence gene expression and how systemic changes in gene expression affect overall health.

## Background

Social and psychosocial factors (henceforth referred to generically as social factors) have consistently been associated with both acute and chronic diseases ranging from the common cold to cardiovascular disease and diabetes [1–4]. Research has now turned to understanding the ways in which these social factors “get under the skin” and cause adverse biological effects that lead to these diseases. One of the mechanisms through which social factors may increase disease risk is via impaired immune system functioning. A large literature has documented associations between low socioeconomic status (SES) in both childhood and adulthood with inflammation in later life [5–8]. Additionally, several factors including discrimination, perceived stress, depression, and cynical distrust have also been associated with altered inflammatory processes [9–13]. Inflammatory processes have in turn been associated with chronic diseases such as cardiovascular disease and diabetes [14–17]. However, the precise biologic mechanisms linking social factors to a heightened inflammatory response have yet to be determined.

Recent studies with limited sample sizes provide preliminary evidence that exposure to adverse social environments is associated with differential gene expression, particularly in immune system related genes. For example, Cole and colleagues report differential expression of 144 genes (209 transcripts) between individuals with high levels of loneliness and low levels of loneliness where more lonely individuals overexpress proinflammatory genes and underexpress antibody synthesis and antiviral immune response genes [18]. This pattern in gene expression differences by social factor has been coined the “conserved transcriptional response to adversity” (CTRA), and has been observed in additional studies [19,20]. Subsequent studies by Cole and others have found similar associations of low SES [21], caregiver stress [22], well-being [23], positive vs. negative affect [24], and grief [25] with expression of immune system related genes. Studies in animals have shown similar findings. Maternal versus surrogate rearing and social group status were associated with differential expression of immune system genes in rhesus macaques [26–28]. In mice, differential expression of inflammatory genes was observed between mice exposed to repeated social defeat and control mice [21].

Despite promising preliminary findings, studies in the emerging field of human social genomics have been limited by small sample sizes and use of multiple cell types (i.e. peripheral blood mononuclear cells) with differing expression patterns. Further, varying thresholds used to identify a statistically significant association across studies make cross study comparisons difficult. To our knowledge, there has not yet been a systematic, large epidemiological study of the relationship between social factors and expression of these previously implicated genes. Using a large population-based cohort with gene expression collected solely in monocytes and three complementary methodologic approaches, we assess whether seven social factors (i.e. childhood SES, adult SES, loneliness, lifetime discrimination, chronic burden, perceived stress, and social support) are associated with expression of genes previously found to be related to social factors. In the first approach, we employ a self-contained gene set enrichment test, Global Analysis of Covariance (ANCOVA), to jointly assess whether expression of any of the investigated genes is associated with each social factor [29]. In the second approach, we use multivariable linear regression to identify whether social factors are associated with the expression of specific genes and apply multiple testing correction [30]. In the third approach, we use elastic net penalized regression as a variable selection procedure to identify the gene expressions that are the most strongly associated with social factors after accounting for correlation across gene expressions [31].

## Materials and methods

### Study sample

The Multi-Ethnic Study of Atherosclerosis (MESA) was designed to investigate risk factors for the development and progression of subclinical cardiovascular disease [32]. The baseline cohort was comprised of 6,814 adults aged 45–84 who self-identified as African-American, Chinese-American, White, or Hispanic and were free from clinical cardiovascular disease. Participants were recruited from six field sites across the United States between 2000 and 2002 (Baltimore, Maryland; Chicago, Illinois; Forsyth County, North Carolina; Los Angeles, California; New York, New York). Four follow-up examinations have been conducted with Exam 5, the fourth follow up, ending in December 2011. The response rate was excellent with 78% participants returning for Exam 5. Each exam consisted of a clinic visit where questionnaires on demographic, psychosocial, and lifestyle factors were administered, and physical assessments including the blood draw needed for genetic analyses were conducted.

Gene expression was assessed in Exam 5 on a random sample of 1,264 individuals [33]. There were three racial/ethnic groups represented in this subsample where 272 participants were non-Hispanic Black, 402 were Hispanic, and 590 were non-Hispanic White. Participants were recruited from four of the six MESA study sites: Forsyth County, North Carolina (49 participants), New York, New York (424 participants) Baltimore, Maryland (317 participants) and St. Paul, Minnesota (474 participants). Non-Hispanic Blacks were recruited mostly from the New York and Maryland sites. Hispanics were recruited from the New York and Minnesota sites. Whites were recruited from all four sites.

### Ethics approval and consent to participate

All participants provided consent to participate in this study. Institutional Review Boards at six field centers (Columbia University, New York; Johns Hopkins University, Baltimore; Northwestern University, Chicago; UCLA, Los Angeles; University of Minnesota, Twin Cities; Wake Forest University, Winston Salem) approved the MESA Study, and data analysis for this manuscript was also approved by the Institutional Review Board at the University of Michigan.

### Social environmental factors

#### Adult socioeconomic status

Highest level of education was collected in Exam 1 and was dichotomized as a measure of adult socioeconomic status. Participants were considered highly educated if they had obtained a college degree or higher.

#### Childhood socioeconomic status

Mother’s and father’s level of education were collected in Exam 2 to proxy childhood socioeconomic status. Childhood socioeconomic status was dichotomized. The participant was considered to have a high childhood socioeconomic status if either parent had at least a high school degree.

#### Perceived stress

Perceived stress was measured in Exam 5 using Cohen’s 4-item Perceived Stress Scale (PSS) [34,35]. The participant was asked whether they felt: unable to control the important things in their life, confident about their ability to handle personal problems, things were going their way, or difficulties were piling up so high that they could not overcome them in the past month. Participants answered on a five point Likert scale corresponding to the answer choices never, almost never, sometimes, fairly often, and very often. A summary measure was created by reverse coding the positive items (items 2 & 3) and summing the items such that a higher value indicated a higher level of perceived stress.

#### Major or lifetime discrimination

Major or lifetime discrimination was collected in Exam 1 and was adapted from the Detroit Area Study [36]. Participants were asked about whether they ever had been unfairly fired or denied a promotion, not hired for a job, treated unfairly by the police, discouraged by a teacher from continuing education, prevented from moving into a neighborhood, or neighbors had made their life difficult [36]. A discrimination score was computed by summing the number of ‘yes’ responses. A higher score indicated higher exposure to major or lifetime discrimination.

#### Chronic burden

Chronic burden was assessed using the Chronic Burden Scale in Exam 3 [37]. Participants were asked whether they had experienced ongoing problems in the following five domains: their own health, health of a loved one, job, relationship, and finances. For affirmative responses, participants were subsequently asked whether this had been a problem for at least 6 months and whether this burden was not very stressful, moderately stressful, or very stressful. To estimate overall chronic burden, we summed the number of domains for which the participant had experienced a chronic burden for at least 6 months and reported that it was either moderately or very stressful. A higher score indicated a higher level of chronic burden.

#### Social support

Social support was measured in Exam 4 using a 4-item scale adapted from the Midlife in the United States (MIDUS) study [38]. The questions asked how much friends and family can be relied upon for help with a serious problem, how open friends and family are to talking about worries, how often friends and family make too many demands, and how often friends and family let the participant down. Possible answer choices included: a lot, some, a little, not at all and were coded as 1, 2, 3, and 4 respectively. Positive items (items 1 & 2) were reverse coded and the sum of the 4 items was calculated to achieve an overall social support score where a higher score indicated greater social support.

#### Loneliness

Loneliness was measured in Exam 4 using a three item scale adapted from the UCLA Loneliness Scale [39]. Participants were asked how often they lack companionship, feel left out, or feel isolated from others. Possible answer choices included: hardly ever, some of the time, and often and were coded as 1, 2, and 3 respectively. A score was created by summing the three items. A higher score indicated a higher level of loneliness.

#### Covariates

Age, sex, and race/ethnicity were self-reported via questionnaire. Study site was indicated by the study team.

### Gene expression

Gene expression data was collected from purified monocytes of 1,264 participants in MESA Exam 5. Detailed methods have been previously described [33]. Briefly, peripheral blood mononuclear cells (PBMCs) were separated within two hours of blood draw using Vautainer cell separation tubes. Monocytes were purified from the PBMCs using anti-CD14-coated magnetic beads. DNA and RNA were extracted using the AllPrep DNA/RNA Mini Kit. The resulting cRNA was hybridized to the Illumina HumanHT-12 v4 Expression BeadChip. This chip has probes for 47,231 transcripts (~31,000 genes), and is designed to assay 12 samples per chip. A stratified random sampling technique was used to assign samples to each chip to avoid biases due to batch, chip, or position.

Preprocessing and quality control steps were conducted to ensure accurate quantification of the gene expression data. Illumina’s proprietary software Genome Studio was used to correct for local background. The remaining preprocessing steps were conducted using Bioconductor packages in R. Since the bead chip has multiple copies of each probe, a bead-type summarization (mean and variance) was produced for each transcript using the *beadarray* package [40]. The negative controls on the array were used to compute the detection p-value. The *limma* package was used for background correction, quantile normalization, log_2_ transformation, and removal of control probes [41]. Quality control criteria for elimination of a transcript included: ‘detected’ expression levels in <10% of MESA samples (detection p-value cut-off = 0.01), probes that contain a SNP, probes with low variance across samples (<10^th^ percentile), and probe overlap with a non-unique region.

### Gene set definition

A literature review was conducted in July 2015 to identify genes whose expression has been associated with social factors in the literature. Much of this work has pointed to differential expression in proinflammatory, anti-viral, and antibody synthesis genes, a profile that has been named the conserved transcriptional response to adversity. To systematically establish a replicable gene set, we limited the search to primary studies that contained the term “conserved transcriptional response to adversity” (CTRA) in Google Scholar or PubMed. This search yielded a total of 63 unique studies. The following inclusion criteria were applied: primary study, gene expression as outcome, and a primate organism. These criteria resulted in 8 studies to be included in the analyses. Since the 2007 study by Cole and colleagues was foundational in describing the conserved transcriptional response to adversity research, we also included this study although it did not explicitly use the term. Therefore, 9 studies were included (Table 1). Of these, 8 were conducted in humans and 1 in rhesus macaques. We combined all the genes that were considered differentially expressed across the studies to compose a single gene set irrespective of whether they were associated with one of the biological processes that defines the CTRA. The HUGO Gene Nomenclature Committee (HGNC) Multisymbol Checker was used to identify all potential synonyms and previous names of genes. Genes that had been withdrawn or did not match to an approved HGNC gene name were excluded. We then used the BioMart ID Conversion tool to match gene names to the transcript identification number on the Illumina HumanHT-12 v4 gene chip. When a gene appeared in more than one study, we only included it once in the gene set (S1 Table). For cases where a gene matched to more than one transcript, we included all transcripts in the analyses. The final gene set consisted of 1,854 transcripts representing 1,305 unique genes.

**Table 1:** Primary Studies of the conserved transcriptional response to adversity in primates.

Study	Exposure	# significant genes	Sample size
Cole, S., et al. (2007)	Loneliness	144	14
Miller, G., et al. (2008)	Caregiving stress	542	21
Cole, S., et al. (2012)	Maternal rearing vs. surrogate rearing	132	9 rhesus macaques
Antoni, M., et al (2012)	Affect Behavioral Therapy	201 90	199
Fredrickson, B., et al (2013):	Hedonic and eudaimonic well-being	53*	84
Powell, N., et al. (2013)	Socioeconomic status	387	60
O’Connor, M., et al (2014)	Non-complicated grief Complicated grief	285 83	63
Wingo, A. and Gibson, G (2015)	Anxiety disorder	631	336
Vedhara, K., et al (2015)	Neuroticism, Extraversion, Openness Agreeableness, Conscientiousness	53*	121

Total number of unique genes (transcripts): 1,305 (1,854)

* In the studies by Fredrickson and Vedhara, 53 genes representing the conserved transcriptional response to adversity were selected a priori to construct a contrast score used in the analysis.

### Global analysis

#### Global ANCOVA

We first assessed the association between each social factor and the entire set of gene expression levels from all previously implicated genes (1,854 transcripts from 1,305 unique genes) using Global ANCOVA [29]. This test has the null hypothesis that no genes in the gene set are associated with exposure status [29,42]. Since the assumption of independent homoscedastic gene expression is unlikely to hold, we used a permutation-based approximation of the F distribution approach in order to assess significance of the Global ANCOVA tests using the strategy described in Hummel 2008 [29].

### Gene level analyses

#### Linear regression

While the global test evaluates the compound hypothesis that at least one gene in a particular set is differentially expressed by social environmental exposure, it is not designed to identify which gene in the set leads to the rejection of the null hypothesis. We conducted subsequent analyses estimating the regression coefficient for each social factor-gene expression relationship. A false discovery rate correction for correlated data of 10% was used to account for multiple testing [30].

#### Elastic net regression

Whereas ordinary least squares approaches such as linear regression identify the best fitting line by minimizing the residual sum of squares (i.e. argmin_β_ |*y* − *Xβ*|_2_), penalized regression methods subject this term to an additional penalty/penalties to improve prediction and interpretation [43,44]. Due to the large number of gene transcripts (*p* = 1,854) that are potentially correlated and modest number of observations (*n* = 1,264) in this study, we use the elastic net penalized regression method to identify the gene transcripts most strongly related to each social factor. Elastic net is designed to conduct variable selection and adjust for correlation among predictors through use of the L_1_ and L_2_ penalties and can be considered as a combination of ridge regression and LASSO [44]. In the present analyses, the two penalties were equally weighted and the tuning parameter was optimally chosen using cross validation. These analyses were conducted using the ‘glmnet’ package in R [45].

### Adjustments

To ensure comparability across the three statistical approaches, we adjusted the social environmental factors and gene expressions for age, sex, study site, race/ethnicity, and chip prior to the main analyses.

### Statistical software

SAS 9.3 and R were used to conduct statistical analyses.

## Results

In the sample of 1,264 MESA participants with available gene expression data, the mean age was 70 years and 51% of the sample was female (Table 2). For the socioeconomic variables, 33% of participants had finished a level of schooling equal to a college degree or higher. Slightly over half of the sample (56%) had either a mother or father achieve at least a high school degree. For most of the social factors, the median scores were close to the minimum score indicating that most of the participants reported low levels of these exposures. There were no significant racial/ethnic differences in these scores except for major or lifetime discrimination (p<0.001). Tukey’s honest significance test, which is used to assess statistically significant differences in means between groups, indicated that Blacks reported higher levels of discrimination than Whites (p<0.001) or Hispanics (p<0.001)[46]. There was not a significant difference between Whites and Hispanics (p = 0.16). The most correlated exposures were loneliness and chronic burden (r = 0.36) and the least correlated exposures were major or life discrimination and perceived stress (r = 0.05) (S2 Table).

**Table 2:** Characteristics of the MESA study sample with gene expression data.

	Total sample (n = 1264)	Non-Hispanic Black (n = 272)	Hispanic (n = 402)	Non-Hispanic White (n = 590)
**Demographics**				
Age. mean (SD)	69.6 (9.4)	69.6 (9.0)	68.4 (9.3)	70.2 (9.5)
Sex (% female)	51%	60%	50%	48%
**Study site *(%)***				
Forsythe County, North Carolina	4%	0.3%	0%	40%
New York, New York	34%	48%	52%	14%
Baltimore, Maryland	25%	51%	0%	30%
Rochester, Minnesota	38%	0%	48%	48%
**Socioeconomic status (%)**				
High education- participant*	33%	27%	13%	49%
High education- either parent**	56%	55%	31%	71%
**Psychosocial factors Median, (Interquartile Range)**				
Loneliness	3 (3–5)	3 (3–4)	4 (3–5)	3 (3–5)
Lifetime Discrimination	0 (0–1)	1 (0–2)	0 (0–1)	0 (0–1)
Chronic Burden	1 (0–2)	1 (0–2)	0 (0–2)	1 (0–2)
Perceived Stress	8 (5–10)	8 (5–10)	8 (5–10)	7 (6–10)
Social support	9 (8–10)	9 (8–10)	9 (8–10)	9 (8–10)

*High education for participant indicates an educational level of a college degree or greater

**High parental education indicates an educational level of a high school degree or greater

### Global analysis

Global ANCOVA gene set enrichment tests assessed the association between each social factor and the entire set of previously implicated genes (1,854 transcripts from 1,305 unique genes) (Table 3). In global analyses, major or lifetime discrimination and chronic burden were significantly associated with the gene set (p = 0.019 and p = 0.047 respectively). Loneliness and adult SES were marginally significant (p = 0.066 and 0.093 respectively). Perceived stress, social support, and child SES were not significantly associated with the gene set.

**Table 3:** Results of the association between social factors with the socially sensitive gene set as assessed via global ANCOVA, linear regression, and elastic net.

Exposure	Global ANCOVA p-value	Linear regression p<0.05	Elastic Net	Overlap between linear regression (p<0.05) and elastic net
Loneliness	0.066	192	74	55
Discrimination	0.019	196	27	27
Perceived Stress	0.611	93	0	0
Chronic Burden	0.047	175	12	2
Social Support	0.662	70	8	7
Adult SES	0.093	117	46	41
Child SES	0.435	63	1	1
# unique transcripts across the seven social factors		738	156	140
# unique transcripts across the four social factors where Global ANCOVA p<0.10		584	150	128

Note: 1,854 gene transcripts investigated in all three approaches

### Gene level analyses

#### Linear regression

In the second approach, we employed multivariable linear regression analyses to identify the specific gene expression transcripts associated with the social factors (Table 3). At p<0.05, we found major or lifetime discrimination to be associated with the greatest number of transcripts (196). The list of transcripts significant at p<0.05 is given in the supplementary material (S3 Table). Generally, the greatest number of significant linear regression results was found for the exposures that were significant or marginally significant in global analyses (i.e. loneliness, major or lifetime discrimination, chronic burden, and adult socioeconomic status). However, no gene transcripts were significant after false discovery rate multiple testing correction.

#### Elastic net

Elastic net regression was used to identify the gene transcripts most strongly associated with each social factor. The number of transcripts selected for each factor is presented in Table 3 and the list of transcripts and corresponding gene names is available in the supplementary material (S4 Table). The greatest number of transcripts was selected for loneliness (74) and the least was selected for perceived stress (0). In total, elastic net regression identified relationships for 156 unique transcripts across the seven social factors. There was very little overlap in implicated transcripts across social factors.

## Discussion

We examined associations between seven social factors and gene expression using a large, multi-ethnic cohort and three complementary analytic approaches. We focused on genes whose expression levels were identified to be socially sensitive in one or more previous studies. Overall, findings suggest that expression levels of a small percentage of genes are associated with exposure to social factors, yet such associations vary by social factor and gene. Although genes included in the *a priori* defined gene set were enriched for immune response genes, expression levels associated with a social factor were found to represent a range of biological functions.

In the first approach, a self-contained gene set enrichment test [42] (Global ANCOVA) was employed to assess the relationship between each social factor with the socially sensitive gene set. Since genes exist in complex biological networks where small changes in gene expression typically occur across many genes, a gene set approach that evaluates groups of genes at a time can reflect the relationships between social factors and gene expression patterning [47]. Further, there is minimal concern for type I error in this analysis as the Global ANCOVA is a single test. In the present study, major or lifetime discrimination and chronic burden were found to significantly associate with the gene set in global analyses. Among the seven investigated factors, these two factors may have yielded significant associations due to a combination of their subjective and enduring nature. The major or lifetime discrimination scale used in the present study was designed to capture impactful experiences that would have long term effects. Assessment of chronic burden was operationalized to be a stressor that was present for six months or more and was perceived to be at least moderately stressful. These findings support prior research in humans and animals that indicate that chronicity and perception of an exposure are important characteristics for health impact, yet adds to the literature by showing such relationships are present at the level of gene expression.[48]

While the global test evaluates the compound hypothesis that at least one gene in a particular set is differentially expressed by social environmental exposure, it is not designed to identify which gene in the set leads to the rejection of the null hypothesis. Linear regression and elastic net regression were subsequently employed to identify the individual gene transcripts associated with each social factor. In the linear regression analyses, there were no statistically significant associations identified after applying false discovery rate correction. However, it is noteworthy that the social factors that were at least marginally significant in the Global ANCOVA (major or lifetime discrimination, chronic burden, loneliness, and adult SES) did yield more significant linear regression results at p<0.05 compared to exposures not significant in global analyses (Table 3). This indicates that linear regression may yield results suggestive of relationships between the social factors and gene expression but is an insufficient approach for reaching a statistically significant threshold potentially due to characteristics such as low power and/or an unknown correlation structure among gene expressions.

There are a number of alternative approaches that handle the curse of dimensionality (i.e. issues that arise in analyzing high dimensional data) by using penalization techniques [49]. Elastic net penalized regression was used to select and jointly estimate the best set of gene transcripts associated with each social factor. The elastic net findings were consistent with the results from the Global ANCOVA and linear regression analyses. The social factors with the most transcripts selected in elastic net (loneliness, discrimination, chronic burden, and adult SES) also had the lower p-values in Global ANCOVA analyses, and most transcripts selected in elastic net were found to have p-values less than 0.05 in linear regression analyses. Across the seven exposures combined, there were a total of 150 unique transcripts selected via elastic net corresponding to 146 unique genes (Table 3). As suggested in previous studies, the gene list was enriched for immune system-related processes. Social factors influence immune system functioning in part through activation of the adrenergic signaling pathway modulates immune cell gene expression [19–21,50–52]. hypothalamic-pituitary-adrenal (HPA) axis where the glucocorticoid-receptor complex has been shown to regulate transcription of immune system-related genes and the sympathetic nervous system (SNS), where the beta Further, ten transcripts were selected for exactly two exposures (3 between adult socioeconomic status and loneliness, 3 between chronic burden and loneliness, 2 between discrimination and loneliness and 2 between support and loneliness). These 10 transcripts corresponded to the following genes: *SLC6A6*, *VNN1*, *SLC22A4*, *IFI6*, *IL12RB1*, *SART3*, *DPP7*, *VPS41*, *SEMA4C*, *and SHMT1*[53]. These genes have a myriad of functions, some with known involvement in the immune response and others without. For example, *IFI6* is involved in interferon gamma signaling, *IL12RB1* is a receptor for interleukin 12, and *DPP7* prevents apoptosis in lymphocytes. On the other hand, *SLC6A6* and *SLC22A4* are transporters, *SART3* is a tumor rejection antigen, *VPS41* is involved in organelle development, *SEMA4C* is involved in axon guidance, and *SHMT1* is an enzyme with involvement in glycine, serine, and threonine metabolism [53]. One transcript was selected for three exposures (loneliness, adult socioeconomic status, and social support). This transcript corresponds to the gene for isocitrate dehydrogenase (*IDH1*), a metabolic enzyme that catalyzes oxidative decarboxylation of isocitrate to 2-oxoglutarate [53]. Mutations in this gene have been associated with different types of cancers, potentially through epigenetic modifications of both oncogenes and tumor suppressor genes [54–56]

### Comparison to previous studies

Among the seven investigated exposures, loneliness and adult socioeconomic status were assessed in previous social genomics studies. In 2007, Cole found there to be at least a 30% difference in expression levels of 209 transcripts (144 genes) between high loneliness and low loneliness individuals [18]. Comparatively, using the same measure of loneliness, the UCLA Loneliness Scale, we found marginal significance with the socially sensitive gene set in global analysis, no significant association after multiple testing adjustment in linear regression analyses, and 74 transcripts to be selected via elastic net. These 74 transcripts mapped onto 74 unique genes, only 6 of which were implicated in the Cole study of loneliness (*CCR2*, *CD79B*, *IL10RA*, *LGALS8*, *RGS1*, and *VNN1*).

Genes identified in a study of the association between adult socioeconomic status and gene expression were also included in our socially sensitive gene set [21]. In a sample of 60 individuals, 387 genes were found to be differentially expressed by 5-year occupational status. In the present study, we used a dichotomized variable of highest level of education as a measure of SES. Education is considered to be a stable measure of SES as education is mostly acquired by young adulthood and is impervious to variations in job status and income [57]. In the present study, the 387 genes found to associate with occupational status were included in our socially sensitive gene set. Our results indicate marginal significance in global analyses, no transcripts significant after multiple testing in linear regression analyses, and 46 transcripts selected via elastic net. These 46 transcripts map onto 44 unique genes. Only 4 of these genes were implicated in the Powell study (*ETV3*, *GBGT1*, *MCEMP1*, *SLC31A2*). In sensitivity analyses, the association between household income as a measure of SES and the gene set was evaluated. However, Global ANCOVA and linear regression tests yielded no significant associations. Elastic net selected two transcripts that were representative of two unique genes FCGR2B, which plays a role in regulation of antibody production, and CDC42, which is involved in cell division [58,59]. FCGR2B was also selected in elastic net analyses for education while CDC42 was not. Comparing the present results for both loneliness and adult socioeconomic status to that of previous studies highlights the difficulty in replicating single gene associations across studies even in the presence of consistent gene set level findings.

Differences in methodological approach likely account for part of the discrepancy between our findings and that of previous studies. Most of the previous studies used an a priori determined effect size definition of differential expression (e.g. 30% difference in expression between low lonely and high lonely individuals)[60]. However, this approach is vulnerable to false positive errors due to the low signal-to-noise ratio inherent in microarray technology [47]. Further, such threshold-based cutoffs do not take into account the highly correlated nature of gene expression [47,61]. Other studies have used a contrast score based on an a priori selected group of genes implicated in prior studies [23], but this score includes only a small subset of the genes investigated in the current analysis which likely accounts for the difference in findings. We improve on these previous methods by employing multiple testing correction designed to account for correlated data in the linear regression models, elastic net as a variable selection method that accounts for multicollinearity, and inclusion of the genes selected for the contrast score along with other genes that have been identified as socially sensitive in the literature.

Differences in sample composition and cell type may also be a reason for the lack of overlap between the present and previous studies. While previous studies tended to be mostly White and from the same geographic region, we had representation from 3 racial/ethnic groups (Hispanic, non-Hispanic Black, non-Hispanic White) from different regions of the country. Our study also had a relatively balanced male to female ratio, whereas the studies used to generate the socially sensitive gene set often were majority female. With a mean age of ~70 years, our sample was older than most of the previous studies. While it is important to understand how these relationships may exist across race and sex, the heterogeneity of our sample likely contributed to the lack of overlap. We did, however, statistically account for these variables in all models. Additionally, differences in cell type may help to account for the different findings between the present and previous studies. Gene expression was collected in monocytes in our study, whereas most of the previous studies used peripheral blood mononuclear cells (PBMCs). PBMCs are a conglomerate of different immune cells types, each of which have different gene expression patterns. Consequently, a statistical association between a social factor and gene expression in one cell type may be muted by null associations in other cell types when PBMCs are used. This concern is reduced in the current study by the sole use of monocytes, one of the more transcriptionally sensitive immune system cell types [62].

### Strengths and limitations

Use of the Multi-Ethnic Study of Atherosclerosis dataset helps to overcome the limitations of previous studies in the literature in the following ways: 1) the large sample size (n = 1,264) compared to previous studies allows increased power to detect differences in gene expression, 2) the comprehensive collection of social factor and gene expression data makes MESA uniquely suited to address the research question, and 3) gene expression was collected in monocytes, an important leukocyte of the innate immune system that prior research indicates is particularly sensitive to social environmental exposures [23,62].

While there were notable strengths to this study, we acknowledge that there were some important limitations. In the interest of systematically identifying social sensitive genes, we limited our literature search to articles which referred to the “conserved transcriptional response to adversity.” In doing this, we may have missed other genes in the literature whose expression is modified by social factors but did not use this term in the published article. A second limitation is the variation in time over which data was collected. Information on social factor exposures was collected at various exams in the MESA study while gene expression was only collected in exam 5. Therefore, there may be time between collection of the exposure and outcome. To minimize the bias this may introduce, we included data from the most recent exam available. Further, since we only had gene expression data available for one time point, we cannot comment on the dynamic nature of the tested relationships.

### Conclusions

Studies that have identified a conserved transcriptional response to adversity were an important stimulus to the emergence of the new field of human social genomics. This area has received widespread attention as it offers a biological mechanism (i.e. gene expression) that has the potential to partly explain the well documented relationships between the social environment and health. Understanding the reproducibility of associations between social and genomic factors will be critical in this precision medicine era where differences in genes, environment, and lifestyles are taken into account to develop tailored prevention and treatment strategies in clinic. This study provides the first large-scale assessment of the social genomics hypothesis in an epidemiological cohort and supports the hypothesis that a set of gene expression levels covaries with social factors. This study is also consistent with previous research in finding that only a small percentage of individual transcript associations are replicable in a separate, diverse human cohort. Methodological approaches that account for complex and potentially unknown correlation structures of gene expression and social factors as employed in the present study are needed to move the field of human social genomics forward.

The future of human social genomics research is promising, and the methods and findings of this study can be used to help guide to future work. Future research should not only validate the findings of the current study but also consider how multiple aspects of an individual’s social environment act in concert to affect gene expression. Secondly, as subsequent studies have been published since our literature review, future work should consider any additional genes identified in those studies. Lastly, studies with longitudinal data are needed to assess temporality of the social factor-gene expression association and effectively examine potential mediating factors including other health conditions (e.g., hypertension and obesity).

## Supporting information

S1 Table List of socially sensitive genes. (CSV)

S2 Table Correlation among continuous social environmental factors. (CSV)

S3 Table Transcripts (genes) with p<0.05 in linear regression analyses by social environmental factor. (CSV)

S4 Table Transcripts (genes) selected via elastic net by social environmental factor. (CSV)

## Abbreviations

*ANCOVA*: Analysis of covariance
*CCR2*: C-C motif chemokine receptor 2
*CD79B*: CD79B antigen (immunoglobulin-associated beta)
*CRP*: C-reactive protein
*CTRA*: Conserved transcriptional response to adversity
*DNA*: Deoxyribonucleic acid
*DPP7*: Dipeptidyl Peptidase 7
*ETV3*: ETS variant 3
*GBGT1*: Globoside alpha-1,3-N-acetylgalactosaminyltransferase 1 (FORS Blood Group)
*HCNC*: HUGO Gene Nomenclature Committee
*HPA*: Hypothalamic pituitary adrenal
*HUGO*: Human Genome Organisation
*IDH1*: Isocitrate dehydrogenase
*IFI6*: Interferon Alpha Inducible Protein 6
*IL10RA*: Interleukin 10 receptor subunit alpha
*IL12RB1*: Interleukin 12 Receptor Subunit Beta 1
*IL-6*: Interleukin 6
*LASSO*: Least absolute shrinkage and selection operator
*LGALS8*: Galectin 8
*MCEMP1*: Mast cell expressed membrane protein 1
*MESA*: Multi-Ethnic Study of Atherosclerosis
*MIDUS*: Midlife in the United States
*OLS*: Ordinary Least Squares
*PBMC*: Peripheral blood mononuclear cells
*PRIM*: Patient rule induction method
*PSS*: Perceived stress scale
*RGS1*: Regulator of G protein signaling 1
*RNA*: Ribonucleic acid
*SAM*: Sympathetic adrenal medullary
*SART3*: Squamous Cell Carcinoma Antigen Recognized By T-Cells 3
*SEMA4C*: Semaphorin 4C
*SES*: Socioeconomic status
*SHMT1*: Serine Hydroxymethyltransferase 1
*SLC22A4*: Solute Carrier Family 22 Member 4
*SLC31A2*: Solute carrier family 31 member 2
*SLC6A6*: Solute Carrier Family 6 Member 6
*UCLA*: University of California-Los Angeles
*VNN1*: Vanin1
*VPS41*: Vacuolar protein sorting-associated protein 41
